# Lyme neuroborreliosis with encephalitis: A rare case

**DOI:** 10.1016/j.idcr.2023.e01704

**Published:** 2023-02-03

**Authors:** Simone Bruhn Rosendahl, Pernille Ravn, Anne-Mette Lebech, Christian Peter Midtgaard Stenør

**Affiliations:** a Department of Internal Medicine, Infectious Diseases Section, Copenhagen University Hospital - Herlev-Gentofte, Herlev, Denmark; b Department of Infectious Diseases, Copenhagen University Hospital – Rigshospitalet, Copenhagen, Denmark; c Department of Neurology, Copenhagen University Hospital Herlev-Gentofte, Herlev, Denmark; d Faculty of Health and Medical Sciences, University of Copenhagen, Copenhagen, Denmark

**Keywords:** Lyme neuroborreliosis, Encephalitis, Neuroinfection, Immunosuppression, MRI

## Abstract

Encephalitis caused by *Borrelia burgdorferi sensu lato* is a rare clinical manifestation of Lyme neuroborreliosis and only in few cases have brain parenchymal inflammation been documented. Here, we present a case of Lyme neuroborreliosis with encephalitis with significant parenchymal inflammation on magnetic resonance imaging (MRI) in an immunosuppressed patient.

## Highlights

• We here report a rare case of Lyme neuroborreliosis with encephalitis.
• The patient was immunosuppressed and presented with uncharacteristic symptoms.
• Magnetic Resonance imaging showed significant parenchymal changes.
• Early diagnosis of this treatable infection is of great importance.

## Background

Lyme neuroborreliosis (LNB) is a tick-borne neurological infection, caused by the spirochetes of the *Borrelia burgdorferi sensu latu* complex.

In Europe, LNB is among the most common bacterial neurological infections and mainly caused by *B. garinii*. In adults, typical nervous system manifestations include radiculoneuritis, cranial neuritis or lymphocytic meningitis. The combination of painful meningoradiculitis, peripheral motor paresis and spinal fluid inflammation is known as the Bannworth triad [1]. Diagnosis of definite LNB by criteria from the European Federation of Neurological Societies (EFNS) [2] requires 1) neurological symptoms compatible with LNB, 2) cerebrospinal fluid (CSF) pleocytosis, and 3) detection of intrathecal *B. burgdorferi* specific IgG and/or IgM antibody synthesis.

Probable or confirmed encephalitis, defined as altered mental state (major criterion) and ≥ 3 minor criteria including new onset of focal neurologic findings, CSF white blood cells (WBC) count ≥ 5/cubic mm and abnormality of brain parenchyma on neuroimaging [3], [4], is a rare manifestation of LNB [5].

## Case presentation

A 74 year-old woman with a history of systemic lupus erythematosus, myasthenia gravis (azathioprine and pyridostigmine treated), osteoporosis and atrial fibrillation was hospitalized four times within a period of three months from September to November. She had been admitted with confusion, paranoid delusions, a weight loss of 15 kg, back pains, history of fever and vomiting suspect of cancer and infection of unknown origin. During these admissions, she was treated with antibiotics for a urine tract infection. She underwent gastroscopy (normal), colonoscopy (showing diverticulosis and three benign polyps), computed tomography (CT) of the neck-thorax-abdomen and ^18^F-fluorodeoxyglucose (FDG) PET/CT (which was normal except for a slightly enlarged spleen).

Previously, the patient was described with a high performance status, without any signs of dementia or known psychiatric diagnosis.

On the last admission in November, central nervous system (CNS) infection was suspected. Neurological examination disclosed disorientation, dysphasia, supranuclear signs with bilateral palmomental reflex, moderate vertical gaze paresis, significant postural tremor with superimposed intermittent myoclonus at the shoulders, elbow and fingers, universal mild hyperreflexia, and bilateral Babinski sign.

Lumbar puncture was performed and empirical intravenous treatment for bacterial meningitis and viral encephalitis was initiated with benzylpenicilline, ceftriaxone, aciclovir and dexamethasone. CSF demonstrated a mononuclear pleocytosis (44·10^6^/L leukocytes, 98 % mononuclear) with a high protein concentration (2.62 g/L), high lactate (4.0 mmol/L) and low glucose (1.4 mmol/L); CSF:Blood glucose ratio was low (0.26) (Table 1). At admittance C-reactive protein (CRP) and white blood cell count were normal.

**Table 1:** Biochemical findings in serum and CSF.

	Result		Reference values
	First lumbar puncture Day of admission	Follow-up lumbar puncture Day 29	
CSF Glucose (mmol/L)	1.4 (blood 5.3)	2.3 (blood 5.0)	2.2–3.9
CSF/blood glucose ratio	0.26	0.46	
CSF Erythrocytes (10^6^/L)	11	4	< 300
CSF Leukocytes (10^6^/L)	44	16	< 5
– Polynuclear (%)	2	3	
– Mononuclear (%)	98	97	
CSF Protein (g/L)	2.62	0.75	0.15–0.50
CSF Lactate (mmol/L)	4.0	Not applicable	1.1–2.4
CSF IgG (mg/L)		259	14–52
CSF Oligoclonal immunoglobulins		Positive	
CSF Neurofilament light polypeptide (ng/L)		3515	< 1850
Glutamate receptor-1-IgG		All negative(CSF and serum)	
Glutamate receptor 2-IgG			
CASPR2-IgG			
GABA-B-receptor 1-IgG			
Glutamate decarboxylase-IgG			
LGI1-IgG			
NMIDAR-IgG			
Biofire DNA/RNA investigations in CSF			
– *Escherichia coli K1*	All negative		
– *Haemophilus influenza*			
– *Listeria monocytogenes*			
– *Neisseria meningitidis*			
– *Streptococcus agalactiae (group B)*			
– *Streptococcus pneumoniae*			
– *Cytomegalovirus*			
– *Enterovirus*			
– *Herpes Simplex Virus 1 + 2*			
– *Humane Herpes Virus 6*			
– *Humane Parachovirus*			
– *Varicella Zoster Virus*			
*B. burgdorferi* intrathecal antibody index			
– IgM	2.946	1.760	
– IgG	2.058	18.513	
*Varicella Zoster* and *Herpes Simplex Virus* intrathecal antibody index	Negative		
*CSF Treponema Pallidum* flagella intrathecal antibodies			
– IgM	0		
– IgG	0		
**Further blood investigations**			
Syphilis test in serum			
*Treponema Pallidum*			
– Flagel IgG	0		
– Flagel IgM	0		
– Phospholipid-Ab (RPR)	0		
– Wasserman-AB	0		
*Mycobacterium Tuberculosis* interferon gamma release assay	Negative		
*Serum Tick Borne Encephalitis Virus* IgM + IgG	Negative		
*Serum Cryptococcus neoformans antigen test*	Negative		

**Fig. 1:**
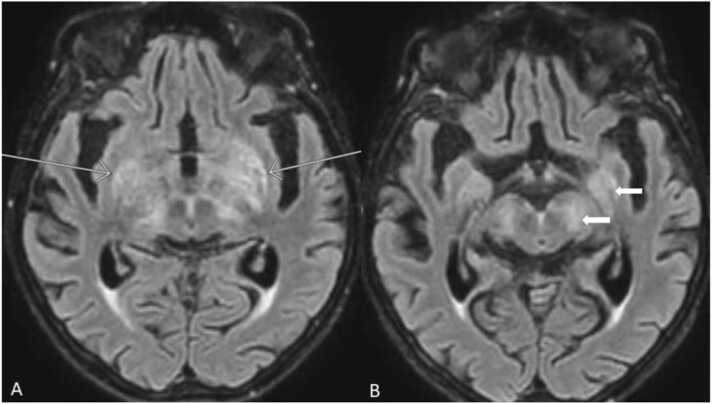
MRI FLAIR with bilateral hyperintensities in A: basal gangliae and thalami (arrows), B: medial temporal lobes and mesencephalon (wide arrows). There was no abnormal contrast enhancement including meninges and no areas of restricted diffusion (not shown). These changes completely resolved on a follow-up MRI (not shown).

Brain MRI performed two days after admission showed symmetric FLAIR hyperintensities in basal gangliae, thalami, medial temporal lobes and mesencephalon without abnormal contrast enhancement (Fig. 1). EEG was without obvious abnormalities.

The patient reported no history of tick-bite, erythema migrans or painful meningoradiculitis. However, approximately three months prior the patient was efficiently treated for a non-itching universal skin rash with a topical steroid and antihistamines.

A diagnosis of LNB was confirmed by detection of a positive *B. burgdorferi* intrathecal antibody index of IgM (2.946) and IgG (2.058; IDEIA™ EIA Test – Oxoid). Serum *B. burgdorferi* IgG antibodies were also positive (125.8 AU/mL; LIAISON® DiaSorin CLIA-test). Neurosyphilis, Tick Borne Encephalitis, cryptococcus, Herpes Simplex Virus (HSV) and autoimmune encephalitis as well as several other viral and bacterial infections that can present as meningitis were ruled out (Table 1).

Benzylpenicilline and dexamethasone were discontinued after one and acyclovir after five days. Antibiotic treatment with high doses of ceftriaxone (4 g daily) was continued until acute bacterial meningitis could be ruled out and the patient was diagnosed with LNB. The patient received a total of 14 days of antibiotic therapy for LNB: seven days of intravenous ceftriaxone (4 g once daily) followed by seven days of oral doxycycline (200 mg b.i.d. for one day and then 100 mg b.i.d. the last six days) with good recovery.

At follow-up two weeks after completed antibiotic therapy for LNB the patient reported clinical improvement and a new lumbar puncture showed decreasing CSF pleocytosis (Table 1). At follow-up 2 months after the LNB diagnosis the patient still experienced cognitive problems such as memory loss. Mini Mental State Examination (MMSE) and Addenbrooke’s Cognitive Test (ACE) were completed with 26/30 and 78/100 points, respectively. Follow-up brain MRI showed complete remission of previous hyperintensities in basal gangliae and thalamus. At 6 months follow-up, the patient had improved but still reported some memory problems (MMSE 29/30 points).

## Discussion and conclusion

We describe a patient with definite LNB by EFNS criteria [2] and encephalitis (definite by international encephalitis consortium criteria (5). A recent Nordic systematic literature review and retrospective cohort study of LNB with encephalitis found a prevalence of encephalitis among LNB patients of 3.3 % (95 % confidence interval 2.2–4.4 %) and a yearly LNB encephalitis incidence of 0.93–1.35 cases/million inhabitants[5]. No specific pattern of brain or spinal cord involvement, like the characteristic involvement of temporal lobes and limbic system in HSV encephalitis, has been found in LNB encephalitis.

In this case, MRI showed hyperintensities in basal gangliae, thalami, medial temporal lobes and mesencephalon and hereby confirmed the diagnosis of encephalitis as the major and ≥ 3 minor criteria were fulfilled [4]. Our findings of significant parenchymal changes on MRI stands out in comparison to the aforementioned Nordic study [5], where varying CT and MRI changes were found in only 20.6 % of patients and were e.g. bilateral white matter changes, frontal left sided edema or vasculitis changes. The minor criteria of abnormal brain parenchyma on neuroimaging suggestive of encephalitis fulfilled here is not well characterized in other LNB cases with encephalitis described in the Nordic study [5]. However, similar MRI changes has been described in a Norwegian study [6].

The long disease course with uncharacteristic symptoms and subsequent diagnostic delay with lumbar puncture first performed three months after debut of symptoms emphasizes the importance of CSF examination in patients with unexplained CNS symptoms. The Nordic study found a median of 14 days from first neurological symptom onset to first hospital contact and from there an additional median of 7 days before initiation of antibiotic therapy. In this case we saw an expected decrease in CSF pleocytosis after intravenous antibiotic treatment and persistent high intrathecal *B. burgdorferi* antibody index as the latter often persists for years after successful antibiotic treatment and therefore cannot be used to monitor treatment efficacy [7], [8]. Interestingly, the CSF glucose level was low at first CSF examination but normalized after antibiotic therapy. In the above mentioned Nordic study median CSF glucose was 3.1 mmol/L whereas a systematic review of LNB related cerebral vascular events reported a median CSF glucose of 1.7 mmol/L [5], [9]. In the latter study median onset of symptoms to diagnosis was 3.5 months, similar to our case. Thus, duration of LNB might exacerbate hypoglycorrhachia as seen in other chronic CNS infections. Additional differential diagnoses must be considered in geriatric patients presenting with acute onset of confusion/altered mental status such as delirium, a wide range of infections (e.g. urinary tract infections, sepsis, pneumonia, CNS infections), stroke/transient cerebral ischemia, etc.

The patient was treated with azathioprine which affects both proliferating B- and T-cells resulting in broad immunosuppression. This could be a contributor to the widespread parenchymal changes seen on MRI and furthermore the atypical symptoms and signs including dysphasia, supranuclear signs, significant postural tremor and not classical signs of early stage LNB with radicular pains and/or peripheral paresis.

A limitation is that the patients was not tested for the presence of *Borrelia miyamotoi*. This tick-borne pathogen has recently been observed in Danish ticks [10] and can cause meningoencephalitis in immunocompromised patients [11]. In addition, patients infected with *B. miyamotoi* can be co-infected with *Borrelia burgdorferi* [12]. However, *B.miyamotoi* is also successfully treated with doxycycline.

In conclusion, we report the, to our knowledge, first case of confirmed LNB encephalitis with significant parenchymal MRI changes in a broadly immunosuppressed patient. Early diagnosis of this treatable infection is of great importance.

## CRediT authorship contribution statement

Simone Bruhn Rosendahl: writing first draft, interpretation. Pernille Ravn: concept, idea to write the case report, feedback, interpretation. Anne-Mette Lebech: feedback, interpretation. Christian Peter Midtgaard Stenør: obtaining patient consent, feedback, interpretation.

## Funding

This research did not receive any specific grant from funding agencies in the public, commercial, or not-for-profit sectors.

## Ethical approval

The patient gave written consent for the publication of this case report.

## Consent

The patient gave written consent for the publication of this case report.

## Conflicts of interest

SBR, PR, CMS: none. AML: outside of the present work, AML reports unrestricted grants for Gilead, speakers honorarium/travel grants/advisory board activity from Gilead/GSK and speakers honorarium/advisory board activity from Pfizer.

## References

[1] Garcia-Monco J.C., Benach J.L. (2019). Lyme neuroborreliosis: clinical outcomes, controversy, pathogenesis, and polymicrobial infections. Ann Neurol.

[2] Mygland A., Ljøstad U., Fingerle V., Rupprecht T., Schmutzhard E., Steiner I. (2010). EFNS guidelines on the diagnosis and management of European Lyme neuroborreliosis. Eur J Neurol.

[3] Granerod J., Ambrose H.E., Davies N.W. (2010). Causes of encephalitis and differences in their clinical presentations in England: a multicentre, population-based prospective study. Lancet Infect Dis.

[4] Venkatesan A., Tunkel A.R., Bloch K.C. (2013). Case definitions, diagnostic algorithms, and priorities in encephalitis: consensus statement of the international encephalitis consortium. Clin Infect Dis: Publ Infect Dis Soc Am.

[5] Knudtzen F.C., Eikeland R., Bremell D. (2022). Lyme neuroborreliosis with encephalitis; a systematic literature review and a Scandinavian cohort study. Clin Microbiol Infect: Publ Eur Soc Clin Microbiol Infect Dis.

[6] Lindland E.S., Solheim A.M., Andreassen S. (2018). Imaging in Lyme neuroborreliosis. Insights Imaging.

[7] Ljøstad U., Skarpaas T., Mygland A. (2007). Clinical usefulness of intrathecal antibody testing in acute Lyme neuroborreliosis. Eur J Neurol.

[8] Kullberg BJ, Vrijmoeth HD, van de Schoor F, Hovius JW. Lyme borreliosis: diagnosis and management. BMJ (Clin Res ed), Vol. 369; 2020, m1041. 10.1136/bmj.m1041 32457042

[9] Garkowski A., Zajkowska J., Zajkowska A. (2017). Cerebrovascular manifestations of Lyme neuroborreliosis–a systematic review of published cases. Front Neurol.

[10] Klitgaard K., Højgaard J., Isbrand A., Madsen J.J., Thorup K., Bødker R. (2019). Screening for multiple tick-borne pathogens in Ixodes ricinus ticks from birds in Denmark during spring and autumn migration seasons. Ticks Tick-borne Dis.

[11] Hovius J.W., de Wever B., Sohne M. (2013). A case of meningoencephalitis by the relapsing fever spirochaete Borrelia miyamotoi in Europe. Lancet.

[12] Boden K., Lobenstein S., Hermann B., Margos G., Fingerle V. (2016). Borrelia miyamotoi-associated neuroborreliosis in immunocompromised person. Emerg Infect Dis.

